# Organizational Rigidity and Demands: A Qualitative Study on Nursing Work in Complex Organizations

**DOI:** 10.3390/nursrep14040242

**Published:** 2024-11-04

**Authors:** Julia van Kraaij, Frits van Merode, Emma Lenssen, Hester Vermeulen, Catharina van Oostveen, RN2Blend Consortium

**Affiliations:** 1 IQ Health Science Department, Radboud University Medical Center, P.O. Box 9101, 6525 EP Nijmegen, The Netherlands; hester.vermeulen@radboudumc.nl; 2 Care and Public Health Research Institute (CAPHRI), Maastricht University, P.O. Box 616, 6200 MD Maastricht, The Netherlands; f.vanmerode@maastrichtuniversity.nl; 3 Maastricht University Medical Centre+, P. Debyelaan 25, 6229 HX Maastricht, The Netherlands; 4 Clinical Research Unit, Radboud University Medical Center, P.O. Box 9101, 6500 HB Nijmegen, The Netherlands; emma.lenssen@radboudumc.nl; 5 Faculty of Health and Social Studies, HAN University of Applied Sciences, 6525 EN Nijmegen, The Netherlands; 6 Spaarne Gasthuis Academy, Spaarne Gasthuis Hospital, P.O. Box 417, 2000 AK Haarlem, The Netherlands; vanoostveen@eshpm.eur.nl; 7 Erasmus School of Health Policy & Management, Erasmus University Rotterdam, P.O. Box 1738, Campus Woudestein, 3000 DR Rotterdam, The Netherlands

**Keywords:** nursing, nursing work environment, nurses roles, hospital care, management, qualitative approaches, work organization, systems thinking

## Abstract

Background/Objectives: The nursing work environment is a critical element in healthcare delivery and a strong predictor of both patient and nurse outcomes. Understanding the complexity and multifaceted nature of this environment is essential for improving nursing practices and optimizing healthcare systems. This study aimed to gain insights into the perceived characteristics of the nursing work environment, considering it as a complex and multifaceted system. Methods: A qualitative research approach was employed, involving 42 semi-structured interviews with 43 nurses and managers from academic, teaching, and general hospitals in The Netherlands. Data were collected between July 2020 and August 2021 through convenience sampling. Thematic coding was conducted to identify key patterns and themes. Results: The findings revealed that nurses demonstrated flexibility and a strong commitment to high-quality care, despite grappling with rigorous organizational tasks and processes. Four key themes emerged: (1) direct patient care as a standard feature of nursing work; (2) nurses’ flexibility for hospital productivity; (3) interdependencies, which decrease autonomous nursing practices; and (4) organizational structures that determine how nurses can shape their work. Nurses found it difficult to balance direct care with broader tasks due to organizational rigidity, revealing a gap between ideal nursing practices and daily reality. Conclusions: This study highlighted the challenges within the nursing work environment, particularly in balancing direct care with organizational demands. Addressing this gap between ideal nursing practice and reality requires a systems approach. This includes autonomous practices, supportive management, and flexible structures, allowing nurses to shape their work and enhance job satisfaction and care quality.

## 1. Introduction

The nursing work environment is crucial for providing high-quality patient care because it substantially impacts numerous critical aspects such as nurses’ job satisfaction and retention [1], patient outcomes [2,3], and organizational performance [4,5]. Nursing work environments are multifaceted, defined as ‘the organizational characteristics of a work setting that facilitate or constrain professional nursing practice’ [6]. Key elements include education and training opportunities for nurses, their involvement in policy-making, relationships with managers and physicians, autonomy, and adequate staffing levels [6,7].

Nurses work in a dynamic healthcare ecosystem, engaging directly with patients, collaborating with other professionals, and navigating various policies, norms, and values. This dynamic nature, constantly changing and evolving, makes the healthcare system unpredictable [8]. In their daily practice, nurses are confronted with situations that demand quick decision-making. They are not only responsible for direct patient care but must also respond to ongoing changes, such as new technologies, updated protocols, and shifts in patient volumes [9]. To effectively manage these challenges, nurses need a clear understanding of the entire healthcare system and recognize how their actions influence other parts of the system, which are also influenced by it. This underscores the importance of acknowledging the dynamic nature of the healthcare system when improving the work environment [10,11]. Improving these environments is crucial for enhancing outcomes for patients, nurses, and healthcare organizations [5].

As healthcare systems worldwide face rising patient demands, staffing shortages, and the need for more efficient, patient-centered care, the pressure to create environments that support and empower nurses has intensified [12,13]. In response to these challenges, healthcare organizations are reconsidering nursing roles, functions, and tasks to better meet the evolving needs of nurses and patients. Some countries divide their nursing workforce based on skills and competencies, whereas others perform so based on educational background. For example, the differentiation of nursing practice, coupled with an increase in the proportion of bachelor-educated nurses, maximizes the skills and expertise of each type of nurse and allows for more efficient and effective patient care delivery, such as reduced length of stay and a lower mortality rate [2,14].

In the Netherlands, differentiated nursing practices among registered nurses have been introduced. Numerous healthcare organizations have restructured their nursing practices to create a more efficient and professional work environment by strengthening nurses’ roles within the organization, boosting the nursing profession’s attractiveness, and elevating the quality of care [15]. This transition extends beyond bachelor- and vocationally trained nurses; it involves a comprehensive transformation of nursing functions, roles, and positions within the organizations as they seek to reinforce the professionalization of nursing [15,16]. These changes highlight the importance of using a systems thinking approach to understand the nursing work environment. This approach views the environment as a complex network of interactions between healthcare professionals, patients, and organizational structures [8]. Systems thinking recognizes how actions in one part of the system can impact the overall performance. By adopting this perspective in healthcare practices, organizations can better understand how individual actions influence the entire system. This leads to stronger connections and greater awareness of the dynamics within the healthcare environment [11].

With the introduction of differentiated nursing practice, a comprehensive understanding of the complex nursing work environment and the necessary changes at all levels within the system is crucial [11,15]. Further research is needed to identify the factors at individual and system levels that enable nurses to thrive in a challenging work environment filled with complexities, uncertainties, and unpredictability [9]. Understanding how significant changes, such as differentiated nursing practices, can be effectively integrated into nurses’ work is crucial. Gaining deeper insights into nursing work environments can lead to improvements in nursing practices, enhance patient care, and optimize healthcare systems [5,17]. Therefore, this study aims to explore the perceived characteristics of the nursing work environment while considering it a complex and multifaceted system.

## 2. Materials and Methods

### 2.1. Design

A qualitative descriptive approach was used to explore the perceived characteristics of the nursing work environment. Qualitative research offers valuable insights into health systems’ complexities, including behaviors and perceptions [11]. By considering the perceptions of nurses and managers, we uncover the elements, strengths, and challenges concerning the nursing work environment, such as the implementation and transition towards differentiated nursing practices. Examining various perspectives enhances understanding of how actions and changes affect different parts of the system [18].

### 2.2. Study Settings and Recruitment

In total, 22 nurses, 13 unit managers, 7 cluster managers, and 1 HR manager (see Box 1 for role descriptions) working in 3 different hospitals were selected by convenience sampling. The hospitals were selected based on their size, geographic location, and type. Characteristics of the selected hospitals and interviewees are shown in Table 1. Cluster managers were asked to distribute an invitation mail among their unit managers and nurses. The invitation mail explained the nature and aim of the research project and how we planned to use the results. Nurses and managers who were interested in participating were invited to schedule an interview by email.

Box 1 **Role descriptions of managers.**

 Unit managers are often referred to as head nurses or team leaders, and they possess a nursing degree. They are responsible for overseeing the operations within a hospital’s nursing department. This involves supervising the nursing staff and managing resources and budget.

 Cluster managers have a broader perspective on nursing management and coordination across various departments. They ensure that the hospital’s vision, policies, and procedures are translated and followed across all nursing departments within their cluster.

 HR managers are involved in workforce management, handling the recruitment, training, and retention of nursing staff to meet both the needs of the departments and organizational goals.

### 2.3. Data Collection

In total, 42 semi-structured interviews with 43 nurses and managers were conducted by JK and EL. Semi-structured interviews involve the use of predefined questions while there is flexibility to introduce new questions as the interviews progress [19]. We chose to conduct individual interviews to explore the personal and detailed thoughts and feelings of the nurses [20]. Two nurses were interviewed together as they both worked in the same department and did not want to be interviewed individually. An interview guide was used during the interviews (Appendix A). Each interview started with the question “What does the patient’s care process from admission to discharge look like?” to identify nurses’ organizational tasks, processes, and dependencies. Further questions were inspired by items from the Practice Environment Scale of the Nursing Work Index (PES-NWI), a tool validated for assessing the quality of the nurses’ work environment [21]. New topics have been incorporated to keep the interview guide up to date with current nursing practice and aligned with a sociotechnical systems perspective [7]. This perspective recognizes the interdependence between the technical (e.g., physical settings), social (e.g., people), and contextual (e.g., social norms and values) aspects of the work environment [22]. Hereby, we focused on interactions and dependencies present in nurses’ daily work. Interviewees were asked about control over their practice setting and teamwork. The interviews were held in Dutch and digitally recorded with the interviewee’s permission. All interviewees were interviewed once, and the interviews took place between July 2020 and August 2021. The day and time of the interviews were arranged to suit the interviewees’ preferences. A total of 14 interviews were held face-to-face and 27 via a video call. Informed consent was obtained from the interviewees before the interviews started. The interviews lasted between 45 and 60 min.

### 2.4. Data Analysis

Data were collected and analyzed in parallel so the interview guide could be modified. The interviews were transcribed verbatim in Dutch by a paid transcription service. A confidentiality agreement was in place, and the company was approved by the Privacy Office at Radboud Academic Medical Center. The transcribed data was randomly checked against the original audio for accuracy.

Atlas-ti version 8.4.20 (ATLAS.ti Scientific Software Development GmbH, Berlin, Germany) was used for thematic coding. Inductive coding was used to formulate themes and stay connected to the data without a pre-existing frame or theory [23]. The six phases of thematic analyses included familiarization with the data, the creation of codes, and the search for and definition of themes.

First, JK, AB, and EL independently coded a subset of the interviews, aiming to gain insights into the characteristics of the work environment. This open coding round labeled all perceived important elements of the work environment. After completing this initial coding round, we discussed the findings and compared the codes. This led to the creation of a preliminary codebook covering thirteen dimensions such as nursing tasks, facilities, work pressure, management roles, nurses’ ambitions, and COVID-19 impact. Following this, JK and AB independently coded the remaining interviews using the agreed-upon codebook, refining and expanding labels. Regular communication was maintained throughout this process to ensure consistency in the application of codes and to address any ambiguities. At the end of the coding process, the team collaboratively reviewed the codes and themes, ultimately formulating four final themes that captured the essential characteristics. These themes were grounded in the data and reflected individual experiences and broader organizational patterns. Finally, the sixth phase involved drafting the report. We synthesized the data and created a narrative that showed how the themes connected to the research questions. JK wrote the initial draft, and all co-authors reviewed and checked the content multiple times.

### 2.5. Ethical Considerations

The local medical ethics review board (Radboud Academic Medical Center, Nijmegen, the Netherlands) declared this study exempt from ethical approval for human subject research (study number: 2019-5992). This decision was made because the study did not involve any interventions that could impact participants’ health or well-being. Participation was voluntary, and confidentiality and anonymity were assured. Interviewees gave written or verbal consent to participate in the interviews and for the interviews to be audio recorded. They were fully informed about the study before giving consent and had the right to withdraw at any time. There were no personal relationships between the interviewees and interviewers. Data were saved under identification numbers according to the rules and legislations of the Radboud Academic Medical Center.

### 2.6. Rigor and Reflexivity

All authors contributed to analyzing and interpreting the findings, as well as finalizing the article. We applied investigator triangulation by involving multiple researchers, which allowed us to integrate diverse perspectives into the analysis. This approach ensured that the findings were validated through a range of viewpoints and areas of expertise [20]. The roles and potential influence on the research process and findings were critically examined and discussed. JK is a nursing science PhD candidate with a background in nursing, business administration, and health and life sciences. CO is a nursing dean and senior researcher and holds a post-academic degree in nursing. FvM is a professor of operations management and logistics in healthcare. HV is a professor of nursing science and clinical epidemiologist. EL works as a research nurse and an MSc candidate in nursing science at the time of the study. AB is a research assistant. Additionally, we conducted member checking by presenting the preliminary results to interviewees at one of the three participating hospitals for validation. This process helped ensure credibility by allowing the interviewees to confirm or clarify our findings.

## 3. Results

Each participating hospital organized their nursing care differently with a variety of tasks and organizational processes. Variations in how differentiated practices were designed and introduced had their own impact on daily nursing practice. Some hospitals clearly distinguished nursing roles and functions based on training, skills, and experiences. In contrast, others adopted a more flexible approach, with all nurses handling similar tasks regardless of their education.

A common form was a differentiation in nursing tasks between regular and specialized nurses. Specialized nurses had received specialty education and were assigned to patients requiring complex care procedures, such as mechanical ventilator support or complex wound management. There were also differences in the distribution of nursing tasks between bachelor-trained and vocationally trained nurses. Bachelor-educated nurses were ‘nurse coordinators’ and vocationally trained nurses were ‘general nurses’. This differentiation was not based on complex patient care but on the execution of transcending and overarching tasks such as coaching colleagues or applying evidence-based practice. In some departments, there was no differentiated nursing practice, nurses were assigned to all types of patients and tasks, despite different educational backgrounds.

All participating nurses were committed to delivering high-quality care and were dedicated to the well-being of their patients. Managers required nurses to be flexible, including adapting to changes in workload distribution and embracing new practices. However, the hospitals strictly adhered to established rules and procedures, and centralized control from the higher management was the norm. We found that, within these systems of numerous interconnections and complexities, the responsibility for addressing patient care challenges often fell on the shoulders of the nursing staff. This tension between expected flexibility and rigid organizational structures manifested in four critical aspects of the nursing work environment. These themes reveal a gap between the ideal vision of the nursing profession and the reality of the current nursing environment. We observed essential elements within the nursing culture in hospitals, which their organizational structures also reflected. This created challenges in introducing improvements within the work environment, such as differentiated nursing practice.

### 3.1. Direct Patient Care as a Standard Feature of Nursing Work

In general, the work environment was perceived as supportive for carrying out routine activities, such as daily recurring technical nursing activities. Both nurses and managers associated the essence of nursing work with the execution of direct patient care and did not link it directly to activities such as quality improvement or evidence-based practice. Nurses identified themselves as being responsible for direct patient care and being meaningful to the patient.

*I would call it a habit. Nurses still think: I do my job at the bedside and other people will execute the tasks beyond that. […] they think: why do I have to be the one to do these suggestions? I’m working with a patient. In my opinion you’re also working with the patient when revising quality documents for which you need the manager or director. However, on a different level. This switch is yet to come.*

*—P25 nurse*

This perspective on the composition of nursing tasks was also seen in the interactions and expectations from colleagues. Nurses collaborated with and supported each other in direct patient care.

*Care workers naturally tend to help, to take over, and to close gaps. When I started working here, I thought this was one of the biggest problems. They just keep walking until they drop. They are not improving processes, but closing gaps because they want to do the right thing for the patient.*

*—P17 cluster manager*

Differentiated nursing practice among bachelor- and vocationally trained nurses appeared to play a role in involving nurses in indirect nursing care activities. Some interviewees pointed out that designating ‘nurse coordinators’ and ‘general nurses’ enhanced the clarity surrounding the allocation of responsibilities and expectations among nurses, as well as their communication with unit and cluster managers. For instance, one unit manager outlined how these roles were distributed, highlighting the nurse coordinator’s (new) pivotal role in coaching and mentoring:

*Our triangle includes a nurse coordinator, an experienced vocationally trained nurse, and an inexperienced vocationally or bachelor-trained nurse. These three take care of a number of patients and the nurse coordinator is assigned to fewer patients than the other nurses. This creates more space for the nurse coordinator to coach colleagues, apply evidence-based practice, or monitor working group processes.*

*—P19 unit manager*

In the organization of both direct and indirect nursing care, it was crucial for nurses to experience a strong sense of support from the hospital. Acknowledgment and appreciation of nurses’ workload by higher levels of management were essential to underscore the value of their contributions. Nonetheless, the one-sided perspective of nursing work, which is centered on bedside care for individual patients, appeared to be deeply rooted, influenced by both organizational expectations and nurses’ own beliefs.

*I made the agreement that on Fridays, I am not available for the ward, but I will be present. I make sure not to wear my white uniform, so it is clear that I am there in a different role. The moment you wear the white uniform, you get caught up even in the smallest things.*

*—P2 nurse*

### 3.2. Nurses’ Flexibility Is Needed for Hospital Productivity

There seemed to be a great need for flexibility from nurses to align capacity with fluctuating demands. Nurse capacity did not seem sufficient to combine direct and non-direct patient care activities. Hospitals were looking for efficient ways to organize nursing work and deploy their nurses. Some interviewees noted the advantages of self-scheduling because it granted nurses more influence in shift allocations.

*If you handle the scheduling yourselves as a team, you allow everyone a degree of influence while setting specific requirements and consequences for not complying […]. I think that it is most effective when individuals get the opportunity to participate in this process.*

*—P41 nurse*

One hospital planned to experiment with the employability of nurses across different departments. In this way, they hoped to balance the demand for care and nursing supplies. The available capacity was calculated based on the average demand for care and patient population. A flexible shell was created where a number of nurses from different departments could be called up to work at another department to answer (temporary) high patient demands.

*We prepare the nursing work schedule based on the average [patient-to-nurse ratio]. Therefore, you have a basic* *team for the average patient population. In case of peaks, you can receive help from the neighbor department. We create clusters and nurses can be lent out within that cluster.*

*—P39 capacity manager*

However, this policy remained theoretical because of growing resistance among nurses. Nurses did not like the idea of working in different teams, and they were unable to consistently apply their expertise in certain departments. Consequently, despite potential gains in efficiency, hospitals hesitated out of concern that nurses might experience reduced job satisfaction and consider leaving the organization. The focus was primarily on productivity and achieving sufficient output, with nurses brought in to meet these demands. Consequently, the specific needs of nurses were sometimes overlooked, and too much flexibility was demanded.

*If you deploy people [red. Nurses] too flexibly, there will be a higher rate of absenteeism. We will end up losing our staff as people do not want that. I get the impression that there is an assumption: “you are a general nurse, widely deployable, so you should do that.”*

*—P42 nurse*

### 3.3. Interdependencies Decrease Autonomous Nursing Practices

The flexibility of nurses was also reflected in the multitude of dependencies observed in their daily work. These work interdependencies were a significant factor influencing the organization and structure of nursing work processes. Nurses highlighted that the differentiation in nursing tasks between regular and specialized nurses increased interdependency in the execution of tasks for both regular and specialized nurses. For instance, certain procedures, like administering chemotherapy treatments or specific dialysis procedures, were exclusively within the purview of specialized nurses. Consequently, different nursing colleagues had to collaborate and coordinate the planning and sequence of care delivery for the same patient during a single shift. These interdependencies created challenges for nurses in managing their individual workload.

*It’s possible that certain procedures may be restricted. For instance, we have a chemotherapy treatment […] once you have completed a certification process, you’re authorized to independently administer it.*

*—P5 nurse*

Nurses were already facing numerous interdependencies with other departments and disciplines. The most frequently mentioned ones pertained to physicians, particularly in decisions related to treatment protocols and patient rounds.

*We experience many dependencies on the physician. If the physician has some delay, then we are stuck in our work.*

*—P8 nurse*

Support processes like quality management or organization management were centrally organized and allocated outside the nursing teams. For instance, the organizational board mandated the use of various questionnaires and measurements to assess the quality of care and nursing practice errors. These outcomes were evaluated outside the department. This centralized organization of processes reflected a rigid structure with decision-making concentrated at higher levels of the hierarchy. This further compounded the nurses’ reliance on their daily tasks and failed to foster professional accountability and ownership. Some interviewees regarded this as self-evident and the best way to organize these activities. Others had a more critical perspective, questioning whether it might be more effective to conduct these evaluations within the team since they were uncertain about how to use the results. The added value of quality management was not recognized and the results did not stimulate self-learning and improvement. One nurse mentioned:

*I am not sure, but I think this is being done hospital wide. I think there are hospital-wide projects, and these results will undoubtedly end up somewhere. Yet I have never seen them, so they are not directly leading to us.*

*—P13 nurse*

Nurses found it challenging to initiate and complete such quality improvement projects because of the rigid structures and their impact on work schedules. Embracing autonomous nursing practice, where nurses assume responsibility for their entire scope of work, could create opportunities to allocate time and resources for quality management projects.

*It is necessary that I indicate in time that I need a day off indirect care to get started with it. If not, I will be scheduled for direct patient care and I know it just won’t happen then. You have to find the space yourself […], you have to take that initiative and figure it out yourself. That you get those days scheduled to get started with it.*

*—P13 nurse*

### 3.4. Organizational Structures Are Leading in How Nurses Can Shape Their Work

To stimulate autonomous nursing practices and the performance of transcending and overarching tasks, nursing teams established specialized working groups focusing on areas such as hygiene prevention, evidence-based practice, and specific diseases. This encouraged nurses to play a more active role in advancing their professional knowledge. However, constraints such as limited time and inadequate financial resources hindered nurses from actively engaging in these working groups. Despite the efforts of unit managers to create space for these activities, it was not always practical or feasible during day-to-day operations as patient-care tasks took precedence. The scarcity of allocated time and the prioritization of direct patient care appeared to obstruct the actual realization of these activities in practice.

*You need to ensure time is provided. It takes some searching, but you can request for non-direct patient care activities […]. We are often short on time, especially if it is a larger project. One day off is not enough. One day every two months is not much and during direct patient care, we do not have the time for it.*

*—P13 nurse*

Staff shortages further complicated these efforts, as nurses assigned to indirect patient care activities were pulled back to the ward when others called in sick, reducing time for quality management.

*There is an area of tension. You want to improve quality in the background, but you also want to deliver quality care on the ward.*

*—P37 nurse*

Both unit and cluster managers found it challenging to delegate responsibilities to nurses when addressing disruptions in the nursing work environment, including tasks like coordinating schedules, bed management, and daily workload management. Several factors contributed to this. Firstly, managers believed that solving these issues on their own would be quicker and more efficient, all the while intending to protect nurses from these tasks and responsibilities, enabling them to focus on providing patient care.

*I sometimes wonder if it has something to do with my leadership style. I like to arrange tasks myself and delegating is not my quality. I want to solve problems as quickly as possible. Everything I do is to protect them. “They are already very occupied, and should I assign them those extra duties?”*

*—P6 unit manager*

Additionally, nurses did not feel responsible or took ownership over the problems, which is illustrated by the following quote:

*It’s always the same nurses who actually have spare time, but are less proactive in taking on tasks [red. non-direct patient care activities].*

*—P7 unit manager*

Tactical decision-making was centralized with the managers and the perspective of nurses was not always considered. Many nurses did not perceive themselves as having a role in this level of decision-making, which further limited their engagement in areas such as the development of healthcare policies and the advancement of organizational objectives. This issue frequently appeared to be caused by an unsupportive organizational structure characterized by formal hierarchies. However, positioning a nurse in a strategic role was described as beneficial by interviewees. This became clear in the following quotation, in which a cluster manager with a nursing background described how nurses have minimal influence because of existing power structures, but he made every effort to represent nurses at the strategic level.

*They do not hold strategic positions. We serve as tactical managers, so it is our task to translate [strategies] to the department […] at the same time, I work to bring nursing care to attention at a strategic level to ensure the ongoing advancement of the nursing profession within the hospital.*

*—P12 cluster manager*

*I hope that step by step we can all say ‘we are nurses in the nursing domain’. That domain comprises expertise and functions [from operational to strategic level] and we all need them to reach a higher level together, to create an own profession. […] Be proud of the profession and what we stand for.*

*—P9 cluster manager*

The dichotomy between the flexibility of nurses and the inflexibility of hospital systems posed difficulties for nursing practice. Both nurses and managers found it difficult to achieve an optimal balance in structures, task allocation, and delegation of responsibilities, as they navigated a complex web of interdependencies. However, identifying and achieving this balance could hold the key to a successful and effective approach to creating a stimulating work environment for nurses.

## 4. Discussion

### 4.1. Main Findings

This study explained several critical aspects of the nursing work environment and the challenges nurses face in their daily practice. Nurses often needed to demonstrate flexibility in their job roles, responsibilities, and collaborative efforts. However, organizational structures were often demanding and inflexible. Hospitals exhibited the characteristics of professional bureaucratic structures [24], a description that is made particularly prominent by the contrast between the inflexibility of hospital systems and the flexibility demanded of nurses. We recognized this dichotomy in four themes that comprise one main focus: advancing and recognizing nursing beyond the routine provision of bedside care in daily practice. The perception of the nursing profession shapes the responsibilities, relationships, task distribution, and structures within the work environment.

The first theme was that direct patient care is a standard feature of nursing work. This demonstrated that nurses were clinically focused and that their work was associated with the direct work environment on their ward. Recognition from peers and supervisors was noted as important in acknowledging nurses’ contributions and workload challenges. Karasek identified this as ‘social support’ within his social job demand-control-support model [25]. The tendency of nurses to take over, close gaps, and directly address unexpected problems seemed effective because short-term patient care could continue [26]. Nurses did not always appear to recognize disruptions in their work environment. This lack of recognition could lead to dissatisfaction, as they may not see opportunities to influence or address these issues. Systems thinking encourages a second-order approach, where nurses look beyond individual problems to consider the broader organizational and systemic factors contributing to the issues [27,28,29]. This would allow for more sustainable improvements in nursing practice and work environments, fostering a culture of continuous improvement and proactive problem-solving.

Rather than only being responsible for taking care of their patients, it is important that nurses are also responsible for questioning their current work practices in the wider organizational context [30]. Our results show that differentiated nursing practice stimulated involvement in transcending routine tasks, fostering the adoption of evidence-based practices, and leading working groups. This shift gave nurses the opportunity to solve problems with a second-order approach and improve their work environment. We relate this to ‘organizing professionalism’ where nursing evolves into a profession with broader influence in departments, organizations, and policy [31].

The second theme was that flexibility is required for hospital productivity. This showed that nurses’ flexibility is dependent on responding to fluctuations in patient care needs. Departments frequently faced staffing shortages, yet nurses were forced to solve these capacity issues. This finding was also emphasized by Wallenburg et al. [32], who characterized nurses as ‘general cargo’, responsible for specific duties and tasks, without acknowledging their individual professionalism, career goals, and values. However, relying on nurse flexibility without addressing the wider workplace factors that affect them could cause more staff to leave [33]. This aligns with the concept of job demands in the Karasek model as nurses are required to respond to fluctuations in patient care needs, which could be a significant stressor in their work [25]. Nurses were also not involved in making decisions on the capability and capacity of care, despite evidence that considering nurses’ judgments improves outcomes for patients, nurses, and healthcare organizations [33]. Nurses having little involvement in decisions about staffing and resources suggests a lack of job control, as they may not have the authority to influence or make decisions about their work environment [25]. When making decisions about nurse staffing, it appeared that completing patient care tasks was not the only significant factor. Elements such as ‘quality work’ or ‘supervising nursing students’ were also important [34]. In addition, previous research has confirmed that hospitals with sufficient nursing resources achieved better health outcomes [2]. This confirms that nurses are valuable assets for improving patient outcomes in healthcare organizations.

Our study underscored the influence of middle managers (unit managers) on the nurses’ work environment [35]. Unit managers are uniquely positioned to reduce power differences, enhance trust, and contribute to a more collaborative decision-making process. Placing nurses in these strategic roles is crucial, as these roles require individuals who do not rely on authoritative power but are engaged and guided by informal influence [36]. However, there seemed to be tension between middle managers’ roles and their ability to facilitate nurses’ autonomy. In line with existing literature, middle managers sometimes possess an overwhelming urge to provide care [37]. They aimed to protect their nurses by solving problems themselves and leaving them to deal with direct patient care, which may prevent nurses from feeling responsible for solving problems or obstacles in their work.

The third theme was that interdependencies reduce autonomous nursing practices. This showed that nurses experience many dependencies, both within nursing teams and from other departments and disciplines. This is well known because changes in nursing tasks have consequences for those both earlier and further along in the patient care process. Consider, for instance, the perioperative preparation undertaken by a nurse and the subsequent postoperative care. A surgeon occupies an intermediary role between those two activities. This is also referred to as reciprocal dependence [24]. The fewer dependencies, the better the nurses can work uninterrupted, which could enhance their control over their practice and their ability to shape their work [38]. We also showed that many quality improvement tasks were assigned to individuals outside the team, leading to a dependence on these external parties and a lack of ownership within the nursing team. This could result in nurses losing the opportunity or inclination to address these issues themselves. Ideally, nursing teams should contain the expertise to handle such matters themselves, making them self-reliant in terms of innovation and problem-solving. This contributes to a culture of continuous quality improvement, which increases the quality of nursing care and job satisfaction among nurses [39].

The fourth and final theme was that organizational structures that determine how nurses can shape their work. This reflected that the limited involvement of nurses in regulatory activities was reinforced by the existing organizational structures and management practices. Organizational structures of hospitals have frequently been criticized for not creating capability and sufficient time for nurses, and for not involving them in organizational strategic decision-making or policy processes [33,40]. In terms of Galbraith’s theory [36], this indicates a need for more self-contained tasks. Greater control over tasks leads to higher efficiency because less information needs to be processed during the execution of these tasks (information processing). Additionally, it is important to consider the existing power dynamics within hospitals as they can significantly impact nursing practice. Mintzberg [24] pointed out, that the phenomenon of professional bureaucracy, along with its associated rules and regulations, is a product of the informal power wielded by staff departments.

### 4.2. Strengths and Limitations

There are a few limitations to this study that should be considered when interpreting and building on the results. First, while efforts were made to include hospitals of various sizes, geographic locations, and types, the sample may not fully represent the complete Dutch healthcare landscape. Despite this, we believe that our findings may highlight trends and processes in other hospitals, both nationally and internationally. Second, the use of convenience sampling in this study may have introduced selection bias as interviewees were selected based on their availability and willingness to participate. Their perceptions might be different from those who did not agree to participate. Third, data were collected from July 2020 to August 2021, during the COVID-19 pandemic. This could have distorted the picture of the actual situation. Longitudinal data collection could have provided a more comprehensive understanding of the evolving nature of the nursing work environment. However, we believe that these dynamic times have put more focus on nursing work and the nursing environment and have encouraged critical reflection. Finally, the nursing work environment is a multifaceted concept that encompasses various viewpoints and interpretations [7], some of which might not have been fully captured in this study. We tried to overcome this potential limitation by creating a topic list based on the PES-NWI with additional topics from other recent studies. In addition, Campbell et al. [41] recently showed that most PES-NWI elements have maintained their significance over time.

### 4.3. Implications for Policy and Practice

Among the diverse fundamental elements of the nursing work environment, acknowledging nursing beyond the everyday delivery of bedside care has received much attention in this study. To improve nursing practice and enhance the quality of care, hospitals should consider strategies to empower nurses and reduce dependencies in their work. This transformation requires a systems thinking approach, recognizing that nursing is part of a larger health system with dynamic interactions across disciplines and other organizational structures [42].

Reducing dependencies could involve creating more self-contained tasks for nurses, thereby decreasing the amount of information that needs to be processed and giving nurses more control over practice [36]. Instead of focusing on (re)organizing relationships and direct work processes, it could be worthwhile to approach the work environment as an interconnected system, emphasizing the importance of collaboration across sectors, organizations, and disciplines. Nurses should be seen as key actors in this system at all levels, contributing to decision-making processes that impact patient care and organizational culture [11].

Although there is a desire to adopt a differentiated approach, existing organizational cultures and structures do not seem ready for this change. Consequently, managers often rely on nurses to bridge this gap by being adaptable and flexible. When transforming nursing care, it is important to acknowledge and recognize the nature of change and to listen to nurses’ opinions. This can be conducted by defining clear roles and responsibilities and by involving nurses in the reorganization of nursing practice [15]. In a stimulating environment, the complexity of nurses’ work needs to be seen and nurses should be involved in making decisions about the design and implementation of new ideas or changes within the organization [43]. Approaching a nursing-shared governance structure could further support this involvement, giving bedside nurses the opportunity to become involved in decision-making, practices, policies, and protocols [39].

### 4.4. Recommendations for Further Research

Further research on the perceptions of other internal stakeholders, such as patients, physicians, members of the hospital management board, or supporting personnel could provide a complete picture of the nursing work environment. This would also provide the opportunity to uncover the root cause of tension between nurses’ flexibility and organizational constraints, along with the different interests at play.

## 5. Conclusions

This study provides insights into the complexities and challenges nurses face in balancing direct patient care with broader healthcare responsibilities. These findings show that nurses often exhibit flexibility and are committed to providing high-quality healthcare, but that they often contend with organizational inflexibility, work dependencies, and a prevailing emphasis on direct patient care. These factors create a tension between the nurses’ desire to engage in broader nursing roles and the constraints imposed on them by their work environment, revealing a gap between the ideal vision and expectations of the nursing profession and the current reality. To address these challenges, it is crucial to view the work environment as a complex system that includes autonomous and proactive nursing practices, supportive management, empowering structures, and nurse involvement in decision-making. Reducing the demand for flexibility from nurses and increasing organizational flexibility may provide nurses with opportunities to design and shape their own work processes, increase their job satisfaction, and eventually improve the quality of their work. Creating a more flexible and collaborative work environment is essential to enhancing nurses’ control over their practice and securing a sustainable nursing workforce for the future.

## Figures and Tables

**Table 1:** Characteristics of hospitals and interviewees.

Hospital Characteristics		*N* ^1^	
Type	Academic	1	
	Teaching	1	
	General	1	
Number of beds	<500	1	
	500–1000	1	
	>1000	1	
Number of nurses	>2000	1	
	1000–2000	1	
	<1000	1	
Interviewee characteristics		*N* ^2^	%
Gender	Male	8	18.7
	Female	35	81.3
Age (years)	<25	1	2.3
	25–34	11	25.6
	35–44	12	27.9
	45–55	9	20.9
	>55	10	23.3
Education level	Vocational	8	18.6
	Bachelor	24	55.8
	Academic	11	25.6
Function	Nurse	22	51.2
	Unit manager	13	30.2
	Cluster manager	7	16.3
	HR manager	1	2.3
Total responses		43	

N ^1^ = number of hospitals; N ^2^ = number of interviewees.

## Data Availability

The data are not publicly available due to the containing information that could compromise the privacy of the research participants.

## Public Involvement Statement

No public involvement in any aspect of this research.

## Guidelines and Standards Statement

The study was designed and executed in accordance with the COnsolidated criteria for REporting Qualitative research (COREQ) checklist [44].

## Appendix A. Interview Guide

*Demographic information:* Age, education level, function
*Main question:* What does the patient’s care process from admission to discharge look like?

- Are these activities integrated in the department?
- How would you describe your degree of responsibility?
- To what extent are you facilitated to practice autonomously? Can you solve your own problems?

*Topics:* Leadership; control over practice setting; adequate authorization and clear chain of command; role clarity

- Are the executing and regulatory activities divided and present within the nursing team? Can the nursing team design their own work?
- Are you involved in decision-making processes about policy, personnel, or organizational processes?

*Topics:* Nurse participation in hospital affairs; task orientation; working according to guidelines; organizational learning; innovation and change readiness; information distribution

- Who is responsible for monitoring organizational (quality) processes? How is performance measured? Who is responsible for quality assurance?

*Topics:* Nurse foundations for quality of care; incident reporting and handling of errors; performance measurement

- Are you satisfied with your work?
- How would you describe your workload?
- Do you have (technical) resources available?
- Is there room for personal development?

*Topics:* Staffing and resource adequacy; structural and electronical resources available; workload; scheduling; career development; personal development; job satisfaction; level of stress; challenging and fun work; physical comfort; internal work motivation; job retainment

- How would you describe your relationship with your colleagues?
- How do these relationships influence your work?
- How would you describe the culture in your department?

*Topics:* Nurse manager ability; support of nurses; collegial nurse-physician relation; teamwork; relational atmosphere; supportive coworkers; respect; open communication; trust; feeling valued; supportive organizational atmosphere; celebrating achievements; conflict management; justice; rewards; safety climate; employees as valuable partners; shared mission, vision; cultural values; patient-centered culture
*Closing question:* If you could sketch your ideal work environment, what’s in it that you’re missing right now?
